# Nurses' Roles and Experiences with Enhancing Adherence to Tuberculosis Treatment among Patients in Burundi: A Qualitative Study

**DOI:** 10.1155/2014/984218

**Published:** 2014-08-19

**Authors:** Marie Carlsson, Stina Johansson, Remy-Paul Bosela Eale, Berthollet Bwira Kaboru

**Affiliations:** ^1^School of Health and Medical Sciences, Örebro University, 701 82 Örebro, Sweden; ^2^International Leadership University in Burundi, P.O. Box 2330, Bujumbura, Burundi

## Abstract

*Background*. In TB control, poor treatment adherence is a major cause of relapse and drug resistance. Nurses have a critical role in supporting patients in TB treatment process. Yet, very little research has been done to inform policymakers and practitioners on nurses' experiences of treatment adherence among patients with TB.* Aim*. To describe nurses' experiences of supporting treatment adherence among patients with tuberculosis in Burundi.* Method*. The study adopted qualitative approach with a descriptive design. A purposive sampling was performed. Eight nurses were selected from two TB treatment centers in Burundi. Content analysis was used to analyze the data.* Result*. According to the nurses, most patients complete their treatment. Educating patients, providing the medication, observing and following up treatment, and communicating with the patients were the key tasks by nurses to support adherence. Causes for interruption were medication-related difficulties, poverty, and patients' indiscipline. Treatment adherence could also be affected by patients' and nurses' feelings. Providing transportation and meals could enhance treatment compliance.* Conclusion*. Nurses are critical resources to TB treatment success. In a poverty stricken setting, nurses' work could be facilitated and adherence further could be enhanced if socioeconomic problems (transportation and nutritional support) were alleviated.

## 1. Introduction

### 1.1. Global Tuberculosis, TB Treatment, and TB/HIV

TB is one of the most spread diseases in the world. TB is an infectious disease, and second to HIV/AIDS it is the greatest killer worldwide due to a single infectious agent. In 2012, 8,9 million people had symptoms from TB and 1,3 million died from the disease. Over 95% of the people that succumb to the disease are from low- and middle-income countries in Asia and Africa. The reasons are the standard of living and the large spread of HIV/AIDS in these areas. However, thanks to the global efforts within the framework of the Millennium Development Goals (MDGs), TB death rate has dropped by 45% from 1990 to 2012. This is partly because of the implementation of the comprehensive “Stop TB Strategy” promoted by the World Health Organization (WHO). Directly Observed Treatment Short-course (DOTS) is the essential element that has presumably saved over 22 million lives. Because of this strategy, the world is on track to achieve the objective of reversing the spread of TB by 2015 [1, 2].

First-line anti-TB drugs have been used for a long time and resistance to the medicines is growing. Multidrug resistant TB (MDR-TB) is caused by bacteria that do not respond to first-line anti-TB drugs. There are medicines to treat MDR-TB but this second-line treatment is more complicated and has harder side-effects on the patient. The drugs have lower effect and are more expensive than the first-line treatment. The options of second-line treatment are limited and recommended medicines are not always available. The second-line treatment includes extensive chemotherapy up to two years which is more costly and can produce severe side-effect reactions in patients. In 2012 about 450,000 people in the world developed MDR-TB [1].

People with reduced immune systems like HIV/AIDS patients are more sensitive to the bacteria and suffer a 30% greater risk of developing an active TB, compared to people without HIV/AIDS. At least one-third of the people with HIV/AIDS also have the TB bacteria active or latent. In combination, the two diseases make the immune system weaken faster. Almost 25% of all deaths among HIV/AIDS-patients are due to TB. Sub-Saharan Africa accounts for largest population with HIV in the world; 1 in 20 adults is living with the disease. Thus, prevalence of HIV/AIDS is one of the largest contributing factors of the spread of TB in that region [1].

### 1.2. TB Treatment Approach: DOTS

Until 50 years ago, there were no medicines to cure TB. But today TB is a curable disease. The treatment of drug-sensitive TB consists of four antimicrobial drugs, information, supervision, and support to the patient by health workers or trained volunteers. Treatment adherence can be difficult without such supervision and support. The majority of TB cases can be cured when medicines are provided and taken properly [1]. The drugs are taken once a day and it is a strict treatment that is ongoing for at least 6 months, but if the patient's sputum is still positive for active TB after 2 months the treatment is extended to 9 months. Inappropriate or incorrect use of antimicrobial drugs or premature treatment interruption can cause drug resistance and the chances of relapse are increased. Also the use of poor quality medicines can be a contributing factor to drug resistance. MDR-TB can be transmitted in the same ways as drug-sensitive TB [3]. DOT involves observing patients during their intake of medication. This is supposed to enhance treatment adherence among patients, to help them take their medicines regularly, and to successfully complete their treatment. This also prevents the development of MDR-TB. Depending on the local conditions, the supervision can vary from case to case. It may be undertaken at a health facility, in the workplace, in the community, or at home. The supervisors have to be approved by the patients and have to be trained and taught by health staff to be able to supervise [4].

Treatment adherence is essential not only from the perspective of the patient as an individual but also from the community level and preventive perspective. It is known that interrupted treatment is a major cause of relapse and multidrug resistant tuberculosis. It is important that the patients treatment adherence is as high as possible so that as many as possible can be cured and that the incidence of TB can be decreased [1]. For many reasons, adherence is not easy to achieve and National Tuberculosis Control Programs (NTPs) have a responsibility to ensure that the health systems are supportive of patients from diagnosis throughout the treatment. Due to their proximity with the patients in environments characterized by shortages of human health for health, nurses are critical health professionals playing an important role in supporting the patients in their treatment process. However, their experiences with respect to this task are rarely investigated. This study is therefore seeking answers to the following questions. What are the nurses' experiences of treatment adherence among patients with TB? What is the main reason for interrupted TB treatment according to nurses? What do nurses do in supporting patients under TB treatment? What can and could nurses do to increase the adherence among patients with tuberculosis?

## 2. Aim

The aim of the study was to identify nurses' roles and experiences in relation to their work with supporting patients under tuberculosis treatment in Burundi.

## 3. Methods

### 3.1. Setting

Burundi is one of the smallest countries in Africa with a surface of about 27800, km^2^ [5]. The population was, in 2012, around 9.8 million people. During 2012 the TB prevalence in the country was 199 per 100 000, which means that about 19 500 people in Burundi suffered from TB (this includes patients with TB and TB/HIV). There were 6711 new cases of tuberculosis and 305 retreatment cases reported. The retreatment cases include relapses, treatment after failure, and treatment after default. New smear-positive and/or culture-positive cases had a success rate of 92% in 2012 and new smear-negative/extra pulmonary 84%, and among the retreatment cases the success rate was 85%. According to WHO, the treatment success rate among TB patients has been increasing since 2005. As to TB/HIV coinfection, 82% of all the patients with tuberculosis in 2012 also reported a known HIV status, which is a relatively high proportion [2].

In Burundi NTP is called The National Leprosy Tuberculosis Program (PNLT). PNLT is structured according to three levels of a health pyramid: peripheral level, intermediate level, and central level. There are a total of 606 health centres in Burundi; 165 of these are Centres for Screening and Treatment (CDTs), of which 138 are functional treatment centres. In addition to peripheral CDTs, there is one centre in Kibumbu which is the National Reference Centre for Multiresistant TB. All multiresistant TB cases confirmed or suspected at the peripheral levels are referred to this national reference centre.

With regard to human resources, health workers of the CDTs are overseen by multidisciplinary supervision teams at District Health Offices (DHO), which coordinate the peripheral level. These teams are supervised by the intermediate level supported by the central level [6]. In 2009 there were a total of 7576 health workers in Burundi, 4241 nurses and midwives, 255 physicians, and 159 laboratory technicians [7]. The peripheral CDTs are staffed by nurses only, whereas the staff at the MDR centre in Kibumbu is made of a majority of nurses, including nutritionists and a few physicians.

### 3.2. Study Design

This is a qualitative study with a descriptive design. Descriptive qualitative studies are common in nursing and the goal of this type of research is to develop a rich understanding of a phenomenon [8]. Qualitative semistructured interviews have been used in this study and the purpose of these types of interviews is to get complex answers with a lot of information [9].

### 3.3. Sampling

A purposive sampling of nurses from two different centers of tuberculosis in Burundi was performed. One center was located in the city of Bujumbura and the other center was located in the country-side in Kibumbu. The center in Bujumbura treated patients with different types of tuberculosis except for patients with multidrug resistant tuberculosis; they were sent to the center in Kibumbu. The latter treated only patients with drug resistant tuberculosis. Contact was taken with the two centers to see if there were any nurses who were interested in participating in the study. In order to be selected, one needed to be qualified nurse (university graduate) and must have been working with TB treatment issues for at least one year, including the year prior to the interview.

A total of eight nurses were included, four nurses from each center. The participating nurses were between 33 and 52 years old; they had been working as nurses between six and 25 years and with tuberculosis between six and 22 years. Two participants were men and six were women.

### 3.4. Data Collection Process

Data has been collected with qualitative semistructured interviews during a two-week period in January 2014. The purposes of qualitative interviews are to understand how the participants are thinking and feeling, what experiences they have, and how their world looks like [9]. The interviews were held in each of the two different TB treatment centers. A guide with questions was used, and the interviews were recorded. A guide of questions can be used to make sure that the authors do not forget any of the topics [9]. Each nurse was interviewed once for 15 to 40 minutes. No back translation of the interview guide was done as the questions were deemed easy to understand, generic, and rather straightforward. Instead, focus was put on the interpreter to ensure she perfectly understood the questions. The interpreter had previous experience of interpretation in qualitative research interviewing. The interviews were conducted in English, with interpretation from English to French. The first two authors were present during the interviews as well as an interpreter. The interpreter was a student from the partner university in Bujumbura. One author held the interview while the other took notes. To start the interview the nurses were asked to describe a typical treatment of TB. This was meant to make them feel comfortable and to feel that the authors were there to learn from them. In the end of the interviews, the author who had been taking notes was able to ask some complementing questions.

The authors who conducted the interviews felt that saturation was reached during the seventh interview, which was conducted at the MDR centre in Kibumbu. It was realized that all the data was redundant and no new information was coming out. The 8th interview was conducted to see if any new insights could emerge, but this was not the case. It was then concluded that saturation had been reached.

### 3.5. Processing and Analysis of Data

The interviews were transcribed before they were analyzed using content analysis approach inspired by Burnard [10]. The purpose of performing a qualitative content analysis is to convert large masses of data into smaller segments and then to put those segments together into meaningful conceptual patterns [8].

Content analysis is suitable for semistructured or open-ended interviews but it is also suggested that the method can be used for more clearly structured interviews. To use this method the interviews have to be recorded and transcribed. The analysis consisted of 14 steps which were the base for the analysis in this study. Through these steps, themes and issues were identified and linked together under appropriate categories and subcategories [11]. To analyze the transcribed interviews the authors followed some of these steps and the interview transcript was broken down into relevant data. The authors read the transcribed data several times during this step and changed the coding to what was most appropriate. This is called open coding. The open coding still contained a lot of information. In the following step, the open coding that contained the same information was organized into broader subcategories which were then summarized together into categories. The authors performed these steps together and discussed what could be relevant names for the subcategories and categories; which codes should be put together into the same categories was also discussed. The interviews were read through repeatedly to make sure that the categories reflected the data [10]. The authors also discussed the collected data during the time of the analysis to get a relation between the small parts and the whole context. After this, another coauthor who did not participate in the interviews read through the material to ensure the validity. To put everything together, the subcategories and categories were marked with different colors, which made it easier to put the information with the same content into the corresponding unit. The categories were constantly checked against the aim of the study to make sure that they answered the purpose.

### 3.6. Ethical Considerations

Ethical approval was given by the National Tuberculosis Control Program in Burundi which is under the Ministry of Health. The participation was voluntary and the nurses were able to choose the time and place for the interviews. The nurses were given verbal information about the study in their local language. The nurses also received information in writing regarding the purpose of the study, their ethical rights, and their participation. All information was handled with confidentiality and the participating nurses have been unidentified in the result. Each nurse has been given a number instead of their names and the numbers do not depend on the order of which the interviews were held. The nurses were told that they could stop their participation at any time without any consequences. The interviews were recorded but after they had been transcribed they were deleted. The nurses were given information about where the result was going to be published and how they would be able to reach it.

## 4. Results

The analysis resulted in four categories and some subcategories (Table 1).

### 4.1. Understanding of Treatment Adherence

#### 4.1.1. Treatment Adherence's Overall Situation

The opinion from all of the interviewed nurses was that the majority of patients with TB do complete their treatment. Some of the nurses explained that it is difficult for the patients to follow the treatment throughout but that most of the patients try their very best to complete.

*“Most of the patients that we have complete the treatment but besides this obviously there are others who do not complete their treatment. Because of different reasons” (Nurse 1).*

#### 4.1.2. Understanding of the Impact of Gender Issues on Treatment

Six of the respondents stated that it is more usual that women follow the treatment better than men. A few reasons were given to explain this difference, including the fact that women are more emotional than men; they care about their families and are afraid of affecting others in their surroundings. Women also show more courage to keep up with the treatment than men.

*“Women are more touched than men. More emotional, because when you tell them they have TB, that they have to take care of this and take the medication, because if you do not you are going to affect other people. So when you tell them they get scared and fear. And that is why they have to take their medication, because at least they care about their families around them. For the men that is not the same, it is different” (Nurse 1).*

#### 4.1.3. Understanding of HIV/AIDS Effects on TB Incidence and Treatment

The prevalence of HIV/AIDS among patients with tuberculosis is increasing according to four of the interviewed nurses. There was a difference in how the nurses explained the treatment adherence among patients suffering from both TB and HIV/AIDS. Some nurses thought that it is easier for those patients to follow the treatment than for the patients suffering only from TB, whereas some nurses believed that it is the other way around. There were a few different explanations to why it is easy for the patients suffering from both diseases to follow the treatment. The explanations included that the treatment makes the patients feel better and therefore they want to continue, that patients have a schedule for when the medicines are supposed to be taken, that the patients only come to collect their medication for HIV/AIDS once a month, and that these patients get help from a certain group of people. The nurses who described that they do not think it is easy for these patients also mentioned different reasons. Five nurses described that these patients have to take a lot of medication. Some also described that the medicines are taken at different times during the day and that the medications might give the patients side effects and make them weaker.

*“It is hard for them because of the medicines. You find that it is too much for one person to take. At times these medicines cause them to have pain, and at some point may become weaker” (Nurse 8).*

#### 4.1.4. Understanding of Prerequisites for Increased Adherence

The participants described what they thought could help patients get better adherence to the treatment. One nurse talked about the importance of visiting patients in their home and that they would need more transportation to be able to do more visits. Some of the nurses also explained that it would be helpful if they could offer some food for the patients in combination with the medicine because the medication increases appetite. It was also described that daily communication between nurses and patients is helpful to increase compliance. One nurse believed that it could be helpful if patients who live far away from the centres could be offered accommodation in case of difficulties to reach the centres every day.

### 4.2. Practical Work to Support Treatment

#### 4.2.1. Enhancing Knowledge through Education and Information to Patients

Four of eight nurses described education and information to patients as something important in their job to support the patients. The patients are informed and educated about the disease, how to behave and how to prevent transmission of the disease to other people. They are also taught about the advantage of good adherence and the risks associated with interrupted treatment. The nurses also educate patients about side effects and that they should not mix medication with substances like alcohol or other drugs. One nurse mentioned that counseling is important and available for patients whenever they want to talk to somebody.

*“I teach these patients, and tell them how they are supposed to behave and how to take their medications and how to prevent themselves from infected others” (Nurse 1).*

#### 4.2.2. Provision of Medication, DOT, and Treatment Followup

To support the patients with their treatment, nurses describe a number of helpful actions. Almost all, seven out of eight nurses, explained that they follow up the treatment to see how it is going, if the patients improve or if the situation is becoming worse, how the patients feel, if they feel any side effects, and also to see if the patients are taking their medication every day. Four nurses said that they give out the medication to the patients and three of them explained that the patients should take the medication in their presence.

*“I follow up and then I make sure that I communicate daily with the patient that I am dealing with to find out if they took the medication and if it had any side effects on the patient. And then I follow up to find out if the medication that I give the patients is being held, if they are improving or if the situation is becoming worse” (Nurse 2).*

One nurse explained that they have an organization of different members outside the centers to help the nurses follow up patients. One nurse also explained that it is important to call the patients if they do not show up to see why and that it is important to visit patients in their homes. Two nurses said that encouraging patients is supporting and one nurse spoke of morale, that you as a nurse should help patients with their morale. One respondent in the center of Kibumbu explained that the patients sign a contract when they arrive; the contract says that they should stay at the center for 9 months and that they should finish the treatment. This nurse believed this to be a supporting factor. In the center of Kibumbu the patients stay for their whole treatment and they get accommodation, food, and medicines. One nurse stated that asking the patients what they want to eat is also a way to encourage them. The nurses believed that this is supporting the patients to follow through their treatments.

#### 4.2.3. Maintaining a Positive Attitude and Relationship with Patients

Four nurses talked about how to receive the patients, that you, as a nurse, should treat all people the same no matter what, rich or poor. You should try to be close to the patients, be calm, and speak normally in every situation even if the patients are not serene. One nurse specifically spoke about loving your patients, that you as a nurse should treat them as any human beings.

*“You try to be closer to the patients… then you have to be cool, because if you react they are also going to react, so you have to be normal when you are talking to them” (Nurse 1).*

### 4.3. Perceived Reasons for Interrupted Treatment

Even though the nurses said that most patients follow through their treatment some also explained that there are those who do not, because of several different reasons.

#### 4.3.1. Food Insecurity

The findings underscored that treatment is demanding in that the patients have to come to the centre every day to pick up the medicines. Two nurses said that the medication causes the patients to have increased appetite and for different reasons this might be a factor that makes them interrupt the treatment. The respondents stated that either the patients could not afford to eat, or they would rather go and look for food instead of going to the centres and take the medicines. It was also common that, due to lack of food, patients would rather stop taking the medication instead of taking the medicine and be even hungrier.

*“Whereby we find that people are making that effort to take their medication but, because it requires them to eat, because when you are taking such kind of medication it causes them to have appetite and then they have the appetite, they are taking the medication but they cannot afford to eat” (Nurse 3).*

#### 4.3.2. Interruption due to Relief from Symptoms

One nurse also pointed out that treatment interruption could be once the medicines relieved the symptoms. When the patients feel better they stop taking the medication.

#### 4.3.3. Poverty

Five of the eight interviewed nurses described that one big reason for interrupted treatment is poverty. The fact that patients are poor leads to consequences for the treatment. Five nurses also spoke of the distance as a cause of interruption. That it is difficult for the patients to reach the centre, they cannot get transportation. Two nurses said that it is more important to provide for the family than to go and get the medication. So they would like to do that before they go to get the medication. One nurse described that it is usual that men are supposed to provide for the family and that this might cause them to interrupt the treatment.

*“In almost every home, the men are the heads of the family. However much you are sick, still people will be looking up to you. So they do not want to waste that energy, first they want to go here and there to maybe get a little bit of money so that they can provide for their family. So they always talk like they first want to get the money and then they will come back and finish…” (Nurse 2).*

#### 4.3.4. Indiscipline

Five nurses described indiscipline as a reason for interrupted treatment and four said that it is men who are often undisciplined. Two nurses explained that there are those types of patients who do not listen to what nurses tell them and that instead they do the opposite of what they are told. One nurse said that even if they explain that the patients should not mix medications with alcohol or other drugs there are people who do not follow the recommendations. According to four nurses, alcohol and other drugs are a big reason for the patients' undisciplined behaviour. Two nurses described that men are more often addicted to alcohol.

*“It is maybe out of ignorance for the men because, for the nurses, we always educate these people and remind them, so the men, they do it out of ignorance. Because maybe you find that most of them are alcohol addicts, you find that when they feel better they think that it is time to stop taking the medicine [and I should] go back and find [my] boos” (Nurse 8).*

Another nurse explained that men are associated with bars. Another reason for interrupted treatment was explained as forced interruption, for example, when patients happen to be imprisoned.

### 4.4. Mixed Emotional Feelings

#### 4.4.1. Nurses' Personal Feelings Combined with Increased Risks of Infection

Three respondents described that nurses' and patients' feelings could be a contributing factor for nurses' difficulties in supporting patients. One nurse said that nurses sometimes get scared when patients are furious. Another nurse said that she felt bad when patients harass or insult her.

*“…when they do not give me a positive attitude or they harass me or they insult me, I would feel bad and at times I would even cry” (Nurse 4).*

There was also one nurse who explained that it is a risk for those who treat the patients. The nurse explained that it is a bigger risk for them when the patients are so sick that they cannot go and get the medication themselves. Then the nurses have to go to the patients rooms and the risk of getting infected is increased. When patients suffer from both HIV/AIDS and TB, this causes an even greater risk and the nurses have to be careful when they treat them.

#### 4.4.2. Nurses' Perception of Patients Feelings

Four nurses explained that they think patients feel discouraged to follow the treatment because of different reasons. Two of these nurses said that poverty is a reason for their lack of motivation. When patients are poor, having no food security and having to come to the centre every day, they are easily discouraged. One nurse described that if patients are seeking help from a hospital, which also treats other diseases, the patients might have to wait for a long time to get help and then they feel discouraged. Two nurses also explained that the patients feel alone and one said that patients might feel that nobody cares about them. Once frustrated, the patients put all their anger on the nurses and no matter how much nurses try to help them they feel that something is missing.

*“…we are dealing with patients who feel alone, it is like they are discouraged, nobody cares about them. So what I do is like, I give them courage, I give them courage, I give them moral” (Nurse 2).*

## 5. Discussion

The result in this study shows that all of the interviewed nurses had the experience that the majority of the patients follow through the treatment for TB. Nurses are indeed at forefront of TB care and they are critical to informing about opportunities and obstacles to patients' adherence to TB treatment [12]. According to Ugochukwu and colleagues [13], one of the critical roles of nurses in Sub-Saharan Africa is to provide health education to communities, care providers, clients, and patients.

This study shows that nurses in Burundi have an expert knowledge of issues surrounding treatment adherence in Burundi. They have both broad understanding of the magnitude of adherence, issues of adherence among TB and HIV coinfected patients, and reasons for treatment default and possible interventions to improve adherence. Nurses in Burundi report that they are involved in practical work of educating patients on TB and its treatment, providing medication (DOT) and related followup, and ensuring the patients are of good morale to complete their treatment. The latest report from WHO showed that 84–92% of the patients in Burundi had a successful treatment in 2012 [1]. Burundian nurses' support to patients is likely to be a contributing factor to this high adherence.

For nurses to sustain patients' knowledge, they need to be well trained themselves. A study conducted in Ethiopia found that 43% of the patients interrupted their treatment. Patients' knowledge of TB and its treatment was poor but treatment interruption was associated with inadequate supervision of health care providers. One suggested solution was to improve training for service providers [14]. Sagbakken et al. [15] are also describing the lack of knowledge among health workers as a reason for low compliance [15]. Knowledge among patients may not only encourage the patients to higher adherence but also better equip patients for better self-care. According to Orem [16], most activities to maintain health have to come from the individuals themselves. It could be easier for the patients to come up with these activities if they had knowledge about their needs. If the knowledge is lacking, self-care will be deficient, implying increased demand for other people to take over parts of the patients' self-care to compensate for patients' limitations.

The purpose of DOT is to lower the risk of self-care deficit, but the concept does not underscore what the patients can do themselves. DOT is contributing to maintaining self-care requisites but this could be perceived as humiliating for the patients. An article on ethical aspects of DOT conducted in Ethiopia and in Norway showed that in both countries patients with TB were deprived of their autonomy and lacked the opportunity to influence the delivery of their own health care. The patients experienced that they had no power to influence how their treatment was organized, even if they had tried. Patients reported issues with their income during TB treatment which was not flexible and did not take the patients daily routines into consideration. Many patients reported that they had difficulties to keep their job due to DOT requirements [15]. In Burundi there were few reports of interrupted treatment and adherence was reported to be increasing. According to the nurses in Burundi, DOT as an approach is in progress and it is helpful in order to help patients complete the treatment. However, some nurses also explained the inflexibility of DOT and that this could make the treatment harder for patients to follow.

Some patients were reported to feel that the distance they have to go every day for the medication is one of the biggest causes for treatment interruption. Communication between patients and nurses is a key to make DOT work. One way of communication is through community organizations or groups. Engaging a community organisation to help the nurses conduct DOT for those who cannot come to the centres every day may be one way of making the treatment under DOT more flexible. In addition, one reason for increased adherence that was mentioned was improved knowledge about TB in the communities. They also believe that the patients' self-care, the nurses' health work, and the community have to work together to make the treatment successful and to lower the spread of TB.

According to Frieden and Sbarbaro [17] interrupted treatment is not affected by socioeconomic status, educational level, sex, race, severity of illness and dosage, or adverse effects; the only factor affecting the treatment success is the band between patients and the health workers [17]. This study's findings show that poverty, drug regimens' requirements with regard to nutrition, poverty, relief from symptoms, patients' indiscipline, and gender related issues are factors which may affect adherence. Even though treatment and medicines are for free, there are still other aspects of the importance of money during the treatment. The nurses reported that lack of food because of patients' poverty may be one reason for interruption. The nurses explained that they tell the patients that they should eat when they take the medicines and also that the medication may cause increased appetite. Nutritional support provided with the treatment could therefore improve compliance. This support was only available at the MDR-TB center of Kibumbu, as per MDR-TB treatment policy. Another critical problem was a logistical one. Nurses expressed the need for transportation means to help them reach defaulting or irregular patients and to liaise with the communities. Such are recurrent programmatic problems in TB control [18].

Indiscipline among patients was also described as a reason for default or incorrect treatment. The patients do not listen to the nurses when they try to teach them about the disease and the treatment and they do not do as they are told. The respondents did not explain what they thought could help these patients apart from listening and trying to make the patients understand the importance of completing treatment. Individually tailored solutions should be identified. Some nurses also described addiction to alcohol as a type of indiscipline, as patients refuse abiding to recommendations of not mixing alcohol and other drugs with the medicine. Burundi is a postconflict country and people have been traumatized in several ways. Alcohol, drugs, and mental health disorders are likely to affect substantial proportions of the population [19].

The result of this study shows that the nurses believe that there is a difference among men and women regarding adherence to the treatment. All nurses reported observing better adherence among women than men. The authors do not know why this could be and it would be interesting to know if it really is like this.

In Burundi there are 4242 nurses and midwives and 19500 people suffering from TB. According to Cailhol and colleagues, Burundi is in need of more human resources [20]. There is also indication that many nurses are working in Non-Governmental Organizations (NGO) where salaries are higher than in the public sector. DOT requires a high number of human resources to make the system work. At the two centers in Burundi where the research was conducted, adherence was described as good, indicating perhaps that nurses were coping with the amount of patients they are dealing with. Surprisingly, none of the nurses described human resources as a factor that affects the adherence.

The nurses in this study explained that the prevalence of HIV is increasing among patients with TB. This is also strengthened by the latest WHO report of TB/HIV [21]. The result in this study shows that the nurses had different thoughts of whether it was easier or harder for patients suffering from both TB and HIV to follow the treatment compared to patients who suffered only from TB. Both groups of nurses had good arguments as to why it was easier or harder for these patients. The authors' opinion is that it may depend on the patients' personality more than on which types of diseases they are suffering from. WHO explains the importance of integration between the tuberculosis program and the HIV program [22]. The nurses explained that they also provided HIV treatment for patients who were suffering from both diseases. Other nurses also described that patients suffering from both diseases got extra help to manage the respective treatments. The authors of this study got the impression that because of this extra help it is equally difficult/easy for both types of patients to manage the treatment.

Nurses try their best in supporting patients with their treatment but the effort simply costs in terms of feelings and risks. Feelings such as fear were described when the patients express anger and when they harass or insult the nurses. Also, caring for patients with transmissible infectious disease raises fear for contamination. The authors believe that this fear could affect the nurses' support to these patients in a negative way and that their receiving attitude is affected by these feelings. Nurses also explained the patients' feelings and that discourage, demotivation, and loneliness among patients could affect their treatment negatively.

This study might have some limitations. Convenience sampling is not a preferred approach even in qualitative studies but in this study this design was used for several reasons [8]. The time for the study was limited and nurses at the two centers were of limited number. In this particular context, the authors believe that this type of sampling provided valuable information that was sought. As to the sample size, eight nurses were deemed sufficient given the rich amount of information provided, especially since the respondents had long experience of working with TB patients.

The question guide was piloted prior to the interviews. It was adjusted to eliminate ambiguous formulations. Reconstructing the questions might have made the data more reliable. The pilot interview was also useful for the interviewers who did not have any prior experiences of conducting qualitative interviews. This enhanced reliability of the data collection. When presenting the results, quotations were used to demonstrate credibility of the data [8]. In order to ensure confirmability of the interpretation of the data, a third person read through the material to make sure that the study reflects what the participants said.

## 6. Conclusion

Nurses constitute a key resource in ensuring patients with TB complete their treatment. They provide a range of services and demonstrate a multifaceted understanding of problems surrounding adherence to treatment. Nurses' work could be made easier if they were equipped with transportation means and if they could provide nutritional support to more patients in need.

### 6.1. Policy Implications and Further Research

- Although adherence in Burundi seems to be increasing and according to the nurses the majority of the patients complete the treatment, there are still many people suffering from the disease and there are people who do not complete their treatment as they should. This study shows some reasons for interrupted treatment and what nurses can and could do to support the patients in Burundi. TB being one of the most spread diseases in several countries in Sub-Saharan Africa, it is suggested that the results from this study are, to a greater extent, transferable to other similar settings.
- Policy makers may consider the aspects mentioned in this study (e.g., transportation facilities, nutritional support, etc.) in the formulation of national and local strategies to enhance adherence to TB treatment. There is a need for robust cost-effectiveness studies to see if investments in transport for nurses, for instance, and in provision of food to patients could lead to reduced incidence of drug-sensitive TB and of MDR-TB and associated costs.
- Further studies are needed in Burundi to explain the factors behind the reported increases in treatment success and increases in adherence rates so that policy makers could strengthen them further. Many nurses complained about workload. They work every day and many report being continuously exhausted. It could be interesting to do further research on the nurses' working conditions, including the number of nurses in relation to the number of patients, to see if this could have impact on patients' treatment adherence. The factors explaining gender differences in adherence to TB treatment are another possible area of further research.

## Figures and Tables

**Table 1:** Presentation of categories and subcategories.

Subcategory	Category
(i) Treatment adherence overall situation	Understanding of treatment adherence
(ii) Understanding of the impact of gender issues on treatment	
(iii) Understanding of HIV/AIDS effects on TB incidence and treatment	
(iv) Understanding of prerequisites for increased adherence	

(i) Enhancing knowledge through education and information to patients	Practical work to support treatment
(ii) Provision of medication, DOT, and treatment followup	
(iii) Maintaining a positive attitude and relationship with patients	

(i) Food insecurity	Perceived reasons for interrupted treatment
(ii) Relief from symptoms	
(iii) Poverty	
(iv) Indiscipline	

(i) Nurses' personal feelings combined with increased risks of infection	Mixed emotional feelings
(ii) Nurses perception of patients feelings	
